# Visualizing Individual Perceptual Differences Using Intuitive Word-Based Input

**DOI:** 10.3389/fpsyg.2019.01108

**Published:** 2019-05-21

**Authors:** Maki Sakamoto, Junji Watanabe

**Affiliations:** ^1^Department of Informatics, The University of Electro-Communications, Tokyo, Japan; ^2^NTT Communication Science Laboratories, Nippon Telegraph and Telephone Corporation, Kanagawa, Japan

**Keywords:** individualization, sound symbolic words, tactile sensation, tactile perceptual space, visualization, material

## Abstract

Numerous studies have investigated the fundamental dimensions of human tactile perceptual space using a wide range of materials. Participants generally touch materials and quantitatively evaluate variations in tactile sensations for pairs of adjectives pertaining to the material properties, such as smooth—rough and soft—hard. Thus, observers evaluate their perceptual experiences one by one in terms of adjective pairs. We previously proposed an alternative method of qualitative evaluation of tactile sensations. Our system can automatically estimate ratings of fundamental tactile properties from single sound-symbolic words. We were able to construct a word-based perceptual space by collecting words that express tactile sensations and applying them to the system. However, to explore individual differences in perceptual spaces, different databases for converting words into ratings of adjective pairs are required for each individual. To address this, in the present study we created an application that can automatically generate an individualized perceptual space by moving only a few words in the initial word-based perceptual space. In addition, we evaluated the efficacy of the application by comparing the tactile perceptual space before and after use.

## Introduction

The tactile modality is important when evaluating objects in daily life such as when selecting clothes or making decisions about consumer products (Na and Kim, 2001; Barnes et al., 2004; Workman, 2010; Rahman, 2012; Nakatani et al., 2013). To obtain a deeper understanding of tactile perception, many psychophysical studies have investigated tactile perceptual space (TPS) based on the physical properties of materials (Yoshida, 1968; Lyne et al., 1984; Hollins et al., 1993, 2000; Picard et al., 2003; Gescheider et al., 2005; Tiest and Kappers, 2006; Yoshioka et al., 2007; Chen et al., 2009; Okamoto et al., 2013). Sensations are often assessed via subjective ratings of perceived material properties made using several pairs of adjective words such as rough—smooth and hard—soft (the semantic differential method; Osgood et al., 1957), and the results are associated with physical properties of the materials (Okamoto et al., 2013). Many studies have used this method to analyze TPS. Our previous study (Doizaki et al., 2017) differed from previous work in that we proposed a method for estimating evaluations of touch using only a single word. Specifically, we constructed a system that can convert a word that intuitively expresses tactile sensations into an information equivalent derived from evaluations of 26 pairs of touch adjectives. This system enabled us to obtain information from 26 adjective scales via single words, instead of asking multiple direct questions.

Utilizing this system, we generated a word-based TPS (Doizaki et al., 2017). The word-based TPS is characterized by the arrangement of words in a space in a way that represents the relationships among tactile perceptual qualities. In general, a detailed vocabulary is important for describing perceptual experiences (Osgood, 1952; Bhushan et al., 1997; Guest et al., 2011), and so analyses of sensory vocabulary may be beneficial in studying human perceptual space (Malt and Majid, 2013). Word-based analysis of tactile perception has been performed for Indo-European languages such as English (Guest et al., 2011) and for African languages such as Siwa, a language spoken in Ghana (Dingemanse and Majid, 2012). In our previous study (Doizaki et al., 2017), we focused on the word class of sound-symbolic words (SSWs) (adjective-like words with sound-meaning associations). In Japanese, SSWs, or onomatopoeia, can describe differences in tactile sensation at a fine resolution. This is because Japanese has more than 300 touch-related SSWs, which is more than twice the number of adjectives that describe touch experiences (Sakamoto and Watanabe, 2013). As in explorations of color naming (Jameson, 2005; Regier et al., 2007), the word-based approach enables a direct and intuitive assessment of TPS, at least to the extent to which the Japanese language can be used to describe perceptual space (see Sakamoto and Watanabe, 2017 for our collection of word-based materials). The system described in our previous study is advantageous in that, as it is based on a database of sound-meaning associations, it can automatically convert any kind of SSW into a score with respect to 26 pairs of fundamental qualities of touch, including roughness, hardness, and warmth. Thus, a word-based TPS can be generated by simply collecting SSWs related to touch, inputting them into the system, and applying principle component analysis to the ratings (the first and second principle components are the x and y axes of the TPS). However, one drawback of our system is that the assessment of individualized TPS necessitates the construction of a new database for each individual via elaborate experiments.

Individual differences are an important issue in many research domains, and the structural and functional characteristics of individual differences in perception (Andrews et al., 1997; Duncan and Boynton, 2003; Kanai et al., 2010; Miller et al., 2010; Rouw and Scholte, 2010), cognition (Washburn et al., 2005; Fleming et al., 2010), motor behavior (Tuch et al., 2005; Johansen-Berg et al., 2007; van Gaal et al., 2011), decision-making (Forstmann et al., 2010), personality (DeYoung and Gray, 2009; DeYoung et al., 2010), and social cognition (Bickart et al., 2011) have been reported. Researchers have also attempted to characterize individual differences in tactile senses (Hollins et al., 1993, 2000; Tiest and Kappers, 2006). For example, Hollins et al. (1993, 2000) investigated the similarities between 17 tactile stimuli. Multidimensional scaling analysis showed that the perceptual space associated with the stimuli was most likely to be 3-dimensional. However, they found that perceptual spaces differed according to each individual. Tiest and Kappers (2006) revealed a higher number of dimensions in haptic material space using a larger number of tactile materials. However, visualizing and comparing individual differences in such material-based TPSs is difficult because these differences largely depend on the variety of materials used in the experiments. The dimensions of a TPS could vary dramatically amongst individuals. To address this in the present study, we developed an application to produce individualized word-based TPSs simply by modifying the arrangement of the SSWs in the initial TPS. This application enables the production of customized word-based TPSs, while maintaining a general geometrical relationship between words. That is, each SSW moves within the geometrical configurations of the general TPS, rather than moving individually.

In Construction of individualized word-based TPSs, we describe the principle and construction of this new application. In Individual evaluation before and after modification of TPS, we validate this application by comparing TPSs before and after use. In Discussion, we summarize the results of our study and discuss future possibilities of our application.

## Construction of Individualized Word-based TPSs

In this section, we describe the principle of a new application that can individualize word-based TPSs simply by moving SSWs within an initial TPS. First, we created a word-based TPS (Collection and Placement of SSWs). Then, we placed reference materials into the TPS (Collection and Placement of Reference Materials). Users can intuitively move the SSWs with reference to the materials, and arrangements of whole SSWs in the TPS can be modulated according to an algorithm (Algorithm to Control Movement of SSWs).

### Collection and Placement of SSWs

For the collection of SSWs, we entered the phrase “xx (onomatopoeia) touch” as a search term in Google on July 6, 2012. We used a computer with Windows 8 Internet Explorer. The top 43 SSWs (yielding ≥ 1,000 search results) were selected for use in the initial word-based TPS (see Table 1). We then input each word into our system, which can convert an SSW into a multidimensional rating of tactile properties, to obtain a rating score from −1 to +1 [see (Doizaki et al., 2017) for details of the system]. Using a database containing associations between Japanese phonemes and their qualitative ratings, we were able to estimate impressions of individual words by analyzing only the component phonemes. Then, we performed principal component analysis on the rating scores of the six fundamental dimensions of tactile properties: “hard–soft,” “rough–smooth,” “bumpy–flat,” “sticky–slippery,” “wet–dry,” and “warm–cold” (Okamoto et al., 2013). We generated the initial word-based TPS using the first and second principle components as the horizontal axis and the vertical axis, respectively. The distances between SSWs in the word-based TPS represent the degree of similarity in the multidimensional ratings.

**Table 1:** 43 SSWs.

sara-sara	kasa-kasa	puru-puru
tsuru-tsuru	syaka-syaka	syari-syari
sube-sube	gunya-gunya	peta-peta
fuwa-fuwa	puni-puni	gishi-gishi
zara-zara	kori-kori	beto-beto
gowa-gowa	butsu-butsu	jyori-jyori
gotsu-gotsu	boko-boko	nume-nume
mochi-mochi	pasa-pasa	tsubu-tsubu
poko-poko	funi-funi	zaku-zaku
beta-beta	puri-puri	syori-syori
moko-moko	kishi-kishi	sawa-sawa
fuka-fuka	fusa-fusa	mosa-mosa
gasa-gasa	chiku-chiku	funya-funya
nuru-nuru	mofu-mofu	
suru-suru	howa-howa	

### Collection and Placement of Reference Materials

**Figure 1:**
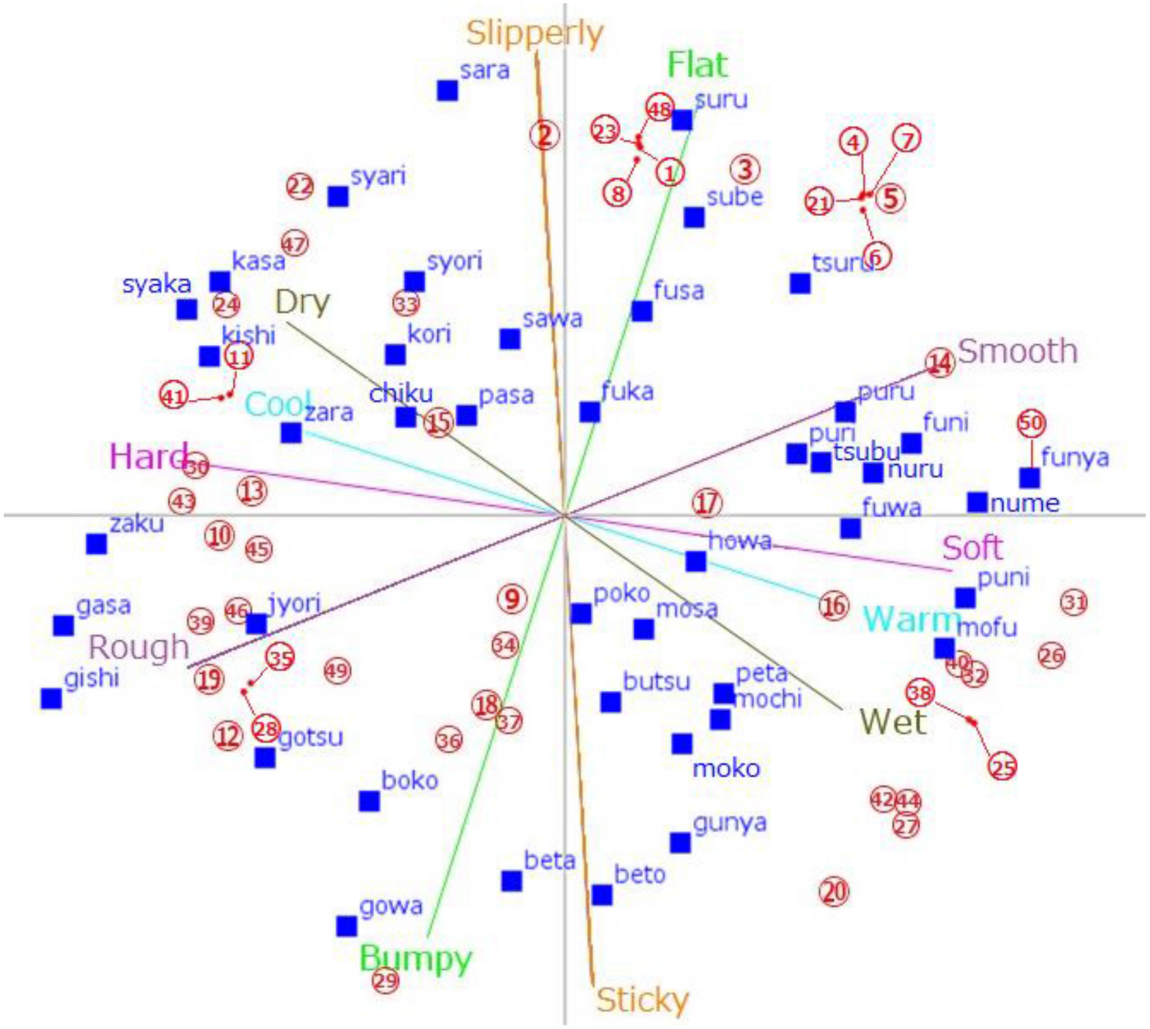
Distribution map of the 43 SSWs and the 50 standardized tactile materials. Blue words indicate SSWs, and red numbers indicate tactile materials. Six tactile scales, “hard–soft,”, “rough–smooth,” “bumpy–flat,” “sticky–slippery,” “wet–dry,” and “warm–cold” are also shown.

To guide users to move the SSWs in the TPS, we used 50 tactile materials (Sakamoto et al., 2013) as references (see Table 2). With respect to the placement of reference materials, participants (six males and four females; mean age 22.8 years) evaluated the tactile impressions of the materials using the semantic differential method. For all participants, we used the same six adjective pairs included in the initial TPS: “hard–soft,” “rough–smooth,” “bumpy–flat,” “sticky–slippery,” “wet–dry,” and “warm–cold”. The participants rated their impressions of each material using a 7-point scale (e.g., −3 = very smooth, 3 = very rough). We normalized the values obtained from each adjective pair with a mean of 0 and variance of 1. Then we placed 50 materials into the word-based TPS using the first and second principle components of the TPS. As a result, 43 SSWs (blue words in Figure 1) and 50 tactile materials (red circled numbers in Figure 1) were placed in the same TPS.

**Table 2:** Fifty tactile materials.

**No**.	**Material**	**No**.	**Material**	**No**.	**Material**	**No**.	**Material**	**No**.	**Material**
1	Paper	11	Stone	21	Iron plate with holes ϕ1 mm	31	Skin-like gel	41	Sandpaper #240
2	Cotton hemp fabric	12	Artificial turf	22	Snake leather	32	Hard slime	42	Soft gel sheet
3	Black alumite sheet	13	Pebble	23	Heat insulator	33	Sandpaper #600	43	Magic tape
4	Acrylic sheet	14	Low-elasticity soft sheet	24	Polystyrene foam	34	Fake fur	44	Paste
5	Glass sheet	15	Iron plate with holes ϕ6 mm	25	Slime	35	Wire-brush	45	Beads
6	Black nickel sheet	16	Adhesive tape	26	Gel gems	36	Cotton	46	Unit turf
7	Aluminum sheet	17	Low-elasticity urethane foam	27	Gel and ball	37	Mouton leather	47	Japanese paper
8	Silk fabric	18	Wool fabric	28	Dot sheet	38	Soft slime	48	Suede (Reverse)
9	Mouton fabric	19	Scourer	29	Vibration-proof rubber	39	Gourd scrubbing brush	49	Carpet
10	Sandpaper #80	20	Vaseline	30	Dry leather	40	Gel sheet	50	Flexible rubber

### Algorithm to Control Movement of SSWs

We constructed an application to generate a TPS for each individual. In the application, users can move the SSWs freely within the TPS. They are expected to designate the placement of the SSWs in the TPS after considering the perceptual relationships between the materials and the SSWs. The application displays the user's TPS on a two-dimensional graphic map. It also stores the individual's username, materials, and initial coordinate data of the SSWs. After the SSWs are moved, the new coordinate data are stored.

#### Moving Location of Words

Here we describe the algorithm that controls movement of the SSWs in the TPS. When a word “A” is moved, another word “B” is also moved according to the distance between the two words. The following equation is used to calculate the influence of moving A on the location of B.

(1) $$\begin{array}{r} \alpha =\text{exp}\left[\frac{-{\left(\text{d}\right)}^{2}}{2{\left(\sigma \right)}^{2}}\right]. \end{array}$$

Specifically, when word A is moved, the influence coefficient α (a value of 0–1), which word B receives, is defined in formula (1), and word B is also shifted by α times the amount that word A moves. The influence coefficient is a Gaussian function, and as the distance (d: pixels) between words A and B increases, the coefficient decreases exponentially. The value of σ in the formula is a constant that defines the range of the influence of word A, and we define the value of σ as 95 in this algorithm. For example, if words A and B were separated by 95 pixels on an 800 × 600-pixel screen, the value of α would be 0.60 and word B would move 60% in the same direction as word A. If the separation were 190 pixels, the value of α would be 0.14, which means that word B would shift by approximately 15%. If the separation were 285 pixels, the value of α would be 0.01, and this would have little effect on the movement of word B.

#### Fixing the Location

In our application, when the user moves an SSW, its location can be fixed by clicking the right mouse button. When moving another word, the influence of fixed words must be considered. To calculate the influence of fixed word C, we use the following equation. Similar to Equation (1), this equation is based on a Gaussian function.

(2) $$\begin{array}{r} \beta =\text{exp}\left[\frac{-{\left(\text{d\_1}\right)}^{2}}{2{\left(\sigma \right)}^{2}}\right]-\text{exp}\left[\frac{-{\left(\text{2d\_2}\right)}^{2}}{2{\left(\sigma \right)}^{2}}\right]. \end{array}$$

**Figure 2:**
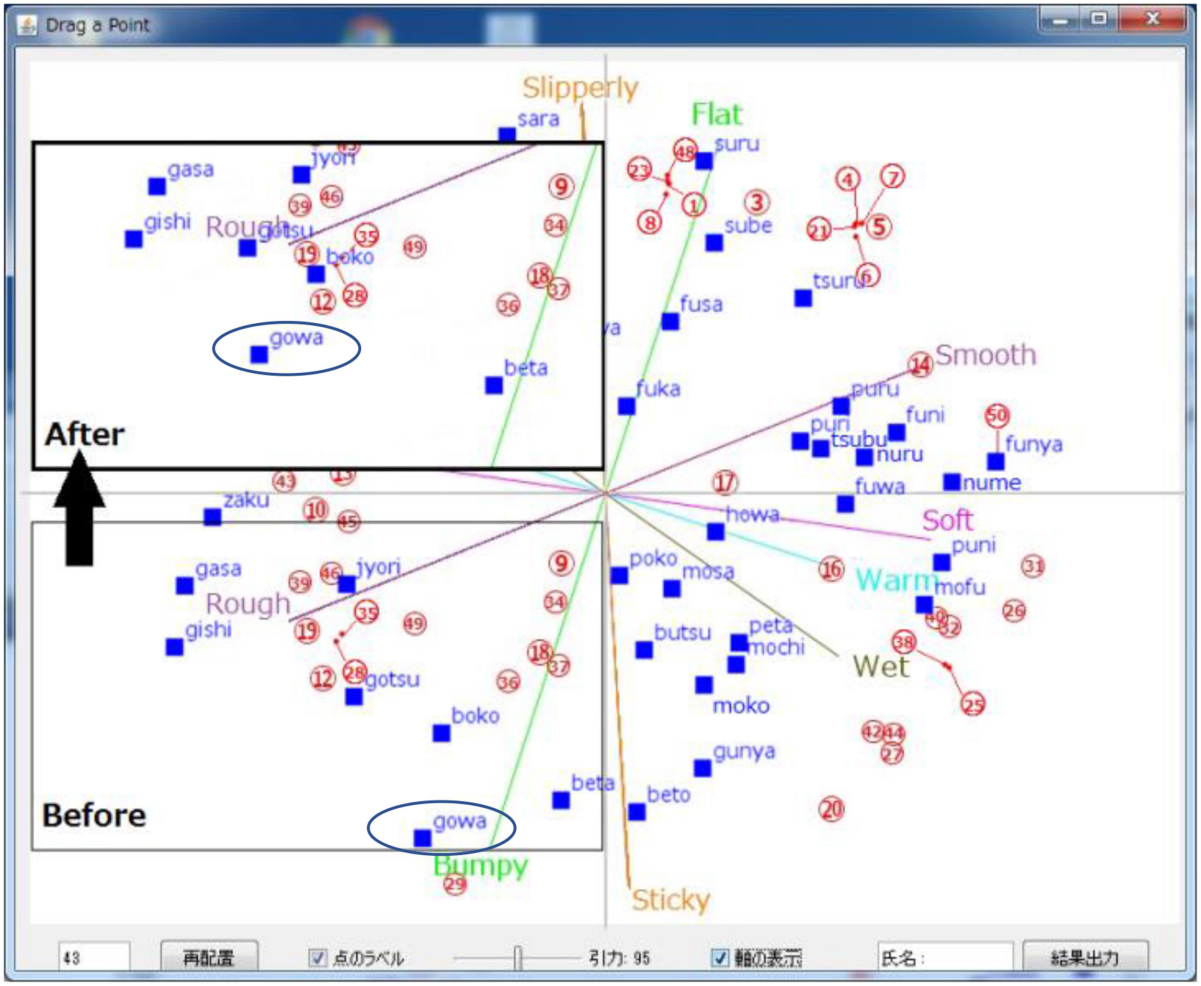
Consequences of a change in the distribution map. In this figure, “gowa-gowa” has been moved to the upper left. Other SSWs, such as “gotsu-gotsu,” “boko-boko,” and “beta-beta,” have been moved according to the influences calculated by Equations (1) and (2).

The influence coefficient β is defined by Equation (2). For example, when word A is moved but word B is fixed, word C is affected by both A and B. The distance (d_1_: pixel) between A and C (the first term) has an influence, as does the distance (d_2_: pixel) between fixed B and C (the second term). The influence of word A is set to be larger than that of word B (the value of the numerator in the second term is 2d_2_). For example, in the case where words A, B, and C are sequentially arranged such that they are 95 pixels apart on the screen, when word A is moved, it influences word B by 0.6 and influences word C by 0.14. Therefore, word C will move 0.46 times in the same direction as the movement of word A. If the distance between words B and C is equal to half the distance between words A and C (d2 = d1/2), the influence coefficient β will be 0 and word C will not move even if word A moves. When the user fixes the locations of words, the influence of these fixed words is added to the second term of β. Users conduct these procedures to change the SSWs in the TPS to match their impressions. Figure 2 shows a state when a certain word is moved in the TPS. In this figure, “gowa-gowa” (circle) has been moved to the upper-left quadrant. Other SSWs, such as “gotsu-gotsu,” “boko-boko,” and “beta-beta,” are then moved according to the influence coefficient calculated by Equations (1) and (2). Differences among individual TPS can be seen by comparing the distribution of SSWs. The number of movements may differ depending on the match between the initial TPS and the TPS for each individual.

## Individual Evaluation Before and After Modification of TPS

We evaluated the efficacy of the application by comparing the matching scores before and after the SSWs in the TPS were moved.

### Participants

Thirty students (undergraduate and graduate) participated in the experiment (15 males, average age 21.7 years). All participants were right-handed and had normal or corrected-to-normal vision, and no known motor-system abnormalities. They had no specialized knowledge about psychophysical experiments and were unaware of the purpose of the experiments. Written informed consent was obtained before the experiment began. The experimental protocol was approved by the Ethics Committee of the University of Electro-Communications and was performed in accordance with the ethical standards outlined in the Declaration of Helsinki.

### Materials

**Figure 3:**
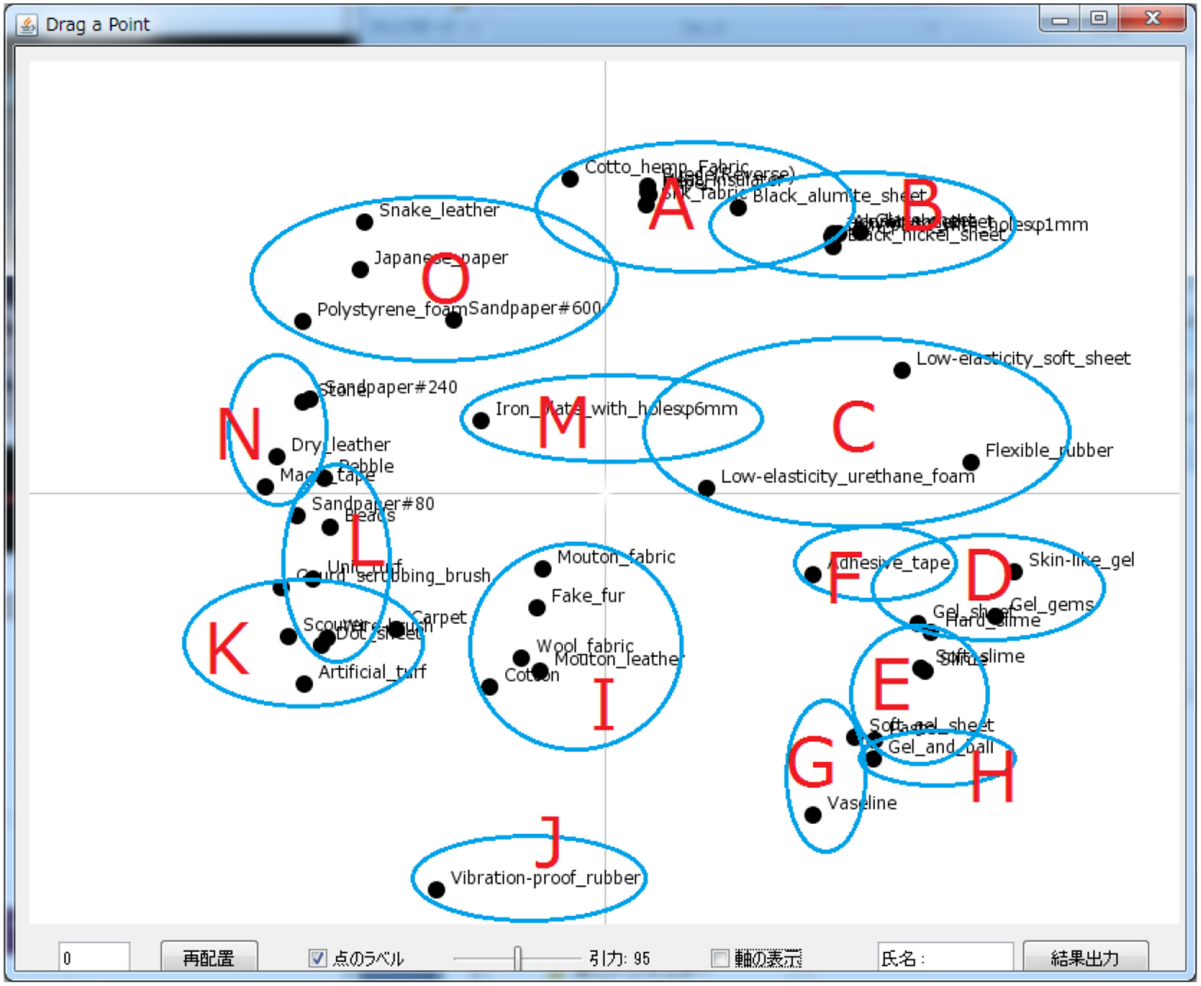
Distribution map visualizing the cluster analysis results.

For the 50 reference materials, we conducted cluster analysis (Ward's clustering algorithm) using the results of the SD ratings that were obtained when the arrangement of the 50 materials was generated. The materials were divided into 15 area groups (see Figure 3), and each area was used to evaluate the matching scores.

### Procedure

To determine which SSWs could be used to modify the initial TPS, we asked the participants to touch the 50 materials and represent their tactile impressions using one SSW for each material. From the reported SSWs (30 people × 50 materials), the 10 most frequently reported were used to move the words: sara-sara (area A), tsuru-tsuru (area B), sube-sube (area B), fuwa-fuwa (area C), puni-puni (area D), peta-peta (middle zone of areas E, F, G, and H), beta-beta (middle zone of areas I and J), boko-boko (middle zone of areas I and J), zara-zara (area M), and chiku-chiku (middle zone of areas L and M). All participants were asked to move each SSW in the order described above and to fix them in the TPS so that they were located closer to, or overlapping, the material number according to their impression when touching the stimuli. Then, the participants confirmed whether the SSW map was appropriate (if not, the participants were allowed to modify it). Next, the matching scores between SSWs and materials were evaluated in each of the 15 material areas before vs. after the words were moved using a 5-point scale (matching: +2, somewhat matching: +1, neither: 0, not matching: −2, somewhat unmatched: −1). After moving 10 SSWs, participants evaluated each area once. The evaluation experiments were performed within 30 min.

### Results

Table 3 shows the average values before (initial TPS) and after (individualized TPS) moving the words. The average value obtained from the initial TPS was 0.62, which represents “somewhat matching.” Moving the words improved the average value of the individualized TPSs to 1.02, indicating that moving the words was an effective method for individualizing the TPS. To compare the average evaluation values before and after moving the words in each material area, we conducted a two-way factorial analysis of variance (ANOVA) with both TPS (before and after moving) and material area as factors. Both main effects were significant [TPS factor: *F*_(1, 29)_ = 20.27, *p* < 0.001; area factor: *F*_(14, 406)_ = 9.69, *p* < 0.001], and the interaction between them was also significant [*F*_(14, 406)_ = 2.71, *p* < 0.001]. These results indicated that although some differences in the effect of the modified areas remained, the matching scores increased after moving the SSWs.

**Table 3:** Average values before (initial TPS) and after (individualized TPS) moving SSWs (*p < 0.05).

Material areas	Average values of initial TPS	Average values of individualized TPS
Group A	1.1	1.533*
Group B	1.2	1.4
Group C	0.767	0.933
Group D	1.033	0.933
Group E	0.1	1.2*
Group F	−0.533	0.333*
Group G	0.6	1
Group H	0.333	0.967*
Group I	−0.1	0.833*
Group J	0.9	1.133
Group K	1.633	1.8
Group L	0.967	0.667
Group M	0.4	0.567
Group N	0.867	0.967

## Discussion

In the present study, we aimed to construct an application to understand and visualize individual differences in the sense of touch. The application is based on a word-based TPS, which consists of SSW sensory vocabulary and reference materials. It enables users to move the SSWs to appropriate locations in the TPS. We evaluated the application by comparing the degree of matching before and after users made modifications to the original TPS. In this section, we discuss the characteristics of the application.

First, the individualized TPSs in this study were word-based TPSs created using SSWs. Numerous studies have investigated TPSs generated based on the physical properties of materials. However, one drawback of material-based TPSs is that they can change depending on the variety of materials used in the experiments. Thus, it can be difficult to directly and intuitively visualize and compare individual differences. Therefore, in the present study, we used SSWs as components of a sensory vocabulary that could correspond with higher tactile dimensions, and implemented an application that enabled users to automatically generate individualized TPS simply by moving SSWs.

Our application can be used to measure perceptual touch characteristics of individuals. Identifying subjective characteristics of touch perception in individuals could enable assessments of differences between product developers and customers. This could be very useful for material and design companies. The quantification of one's tactile experience could also enhance awareness of their own touch perception, thus helping individuals to choose more suitable goods and products. If you want to design products in a specific field, you need to use words and materials of that field.

Our application may be effective in visualizing age-based inter-individual differences, for instance, between younger and older groups of participants. Individual differences in touch might be influenced by age because function in main sensory modalities has been found to decline with age (Victor and Ropper, 2001; Wickremaratchi and Llewelyn, 2006). This may be related to the gradual decline in the number of cells and fibers in the central and peripheral nervous system (Bolton et al., 1966; McLeod, 1980; Schimrigk and Ruttinger, 1980; Katzman and Terry, 1983; Gescheider et al., 1994a; Stevens and Patterson, 1995). Moreover, older people are significantly less sensitive to mechanical stimuli, and their tactile, vibration, pain, and temperature thresholds are significantly higher (Thornbury and Mistretta, 1981; Kenshalo, 1986; Tucker et al., 1989; Gescheider et al., 1994b, 1996; Goble et al., 1996; Verrillo et al., 2002).

Our application might also be used to assess abnormal TPSs in patients with diverse pathologies that affect tactile perception, such as chronic hemiparetic stroke (Ahn et al., 2016) and neurodevelopmental disorders (Cascio, 2010). In the design of new therapies to enrich TPS (e.g., by the addition of tactile noise (Collins et al., 1996; Manjarrez et al., 2003; Mendez-Balbuena et al., 2012), changes in patients' TPSs could be measured periodically during rehabilitation treatment.

Unfortunately, one limitation of our current application is that it is only available in Japanese because the application was constructed using Japanese SSWs. However, the proposed method is likely applicable to other languages such as Siwa in Africa and Basque in Europe, which have SSWs that express tactile sensations.

In summary, individualized word-based TPSs could provide essential information for choosing products or customizing product designs, and may facilitate the assessment of age-related changes in TPS in healthy people, as well as rehabilitation in those with disorders that affect tactile perception.

## Ethics Statement

Written informed consent was obtained before the experiment began. The experimental protocol was approved by the Ethics Committee of the University of Electro-Communications and was performed in accordance with the ethical standards outlined in the Declaration of Helsinki.

## Author Contributions

MS and JW conceived the experiments and discussed the algorithm of the system. MS performed the experiment and analyzed data. All authors discussed and interpreted the results and contributed to drafts of this paper.

### Conflict of Interest Statement

JW is employed by NTT Communication Science Laboratories, Nippon Telegraph and Telephone Corporation, as a research scientist conducting basic scientific research on human sensory processing. There are no patents, products in development or marketed products to declare. This does not alter the authors' adherence to the journal's policies on sharing data and materials. The remaining author declares that the research was conducted in the absence of any commercial or financial relationships that could be construed as a potential conflict of interest.
